# A Practical Guide to Using CV Analysis for Determining the Locus of Synaptic Plasticity

**DOI:** 10.3389/fnsyn.2020.00011

**Published:** 2020-03-27

**Authors:** Jennifer A. Brock, Aurore Thomazeau, Airi Watanabe, Sally Si Ying Li, P. Jesper Sjöström

**Affiliations:** ^1^Centre for Research in Neuroscience, Brain Repair and Integrative Neuroscience Program, Department of Medicine, The Research Institute of the McGill University Health Centre, Montreal General Hospital, Montreal, QC, Canada; ^2^Integrated Program in Neuroscience, McGill University, Montreal, QC, Canada; ^3^Solomon H. Snyder Department of Neuroscience, Johns Hopkins University School of Medicine, Baltimore, MD, United States

**Keywords:** long-term plasticity, long-term potentiation, long-term depression, spike-timing-dependent plasticity, paired recordings, monosynaptic connections, electrophysiology

## Abstract

Long-term synaptic plasticity is widely believed to underlie learning and memory in the brain. Whether plasticity is primarily expressed pre- or postsynaptically has been the subject of considerable debate for many decades. More recently, it is generally agreed that the locus of plasticity depends on a number of factors, such as developmental stage, induction protocol, and synapse type. Since presynaptic expression alters not just the gain but also the short-term dynamics of a synapse, whereas postsynaptic expression only modifies the gain, the locus has fundamental implications for circuits dynamics and computations in the brain. It therefore remains crucial for our understanding of neuronal circuits to know the locus of expression of long-term plasticity. One classical method for elucidating whether plasticity is pre- or postsynaptically expressed is based on analysis of the coefficient of variation (CV), which serves as a measure of noise levels of synaptic neurotransmission. Here, we provide a practical guide to using CV analysis for the purposes of exploring the locus of expression of long-term plasticity, primarily aimed at beginners in the field. We provide relatively simple intuitive background to an otherwise theoretically complex approach as well as simple mathematical derivations for key parametric relationships. We list important pitfalls of the method, accompanied by accessible computer simulations to better illustrate the problems (downloadable from GitHub), and we provide straightforward solutions for these issues.

## Introduction

Synapses transform and transmit information between neurons in a dynamic manner. This activity-dependent capacity to modify the strength of connections between neurons—termed synaptic plasticity—is widely believed to underlie information storage (Bliss and Collingridge, 1993; Malenka and Bear, 2004; Nabavi et al., 2014) as well as circuit remapping during development (Katz and Shatz, 1996; Cline, 1998; Song and Abbott, 2001).

There has been considerable disagreement regarding the locus of expression of long-term plasticity, that is whether the long-term modifications that underpin enduring changes in synaptic efficacy are primarily located presynaptically—through alterations to neurotransmitter release properties—or postsynaptically—through modifications to the number and/or responsiveness of postsynaptic receptors (Lisman, 2003; MacDougall and Fine, 2014; Padamsey and Emptage, 2014). Much of this earlier divisiveness stemmed from the difficulty in analyzing central synapses (Bliss, 1990; Redman, 1990; Korn and Faber, 1991) using classical methods that were developed in the context of the neuromuscular junction (Del Castillo and Katz, 1954). For brevity, the structural and functional differences between the neuromuscular junction and central synapses are not stated here, as they have been reviewed in detail before (Bliss, 1990; Redman, 1990; Sanes and Lichtman, 1999).

After decades of debate, it is now generally accepted that either pre- or postsynaptic mechanisms can support the expression of long-term plasticity (Sheng and Kim, 2002; Malenka and Bear, 2004; Castillo, 2012). In fact, there is also evidence for both pre- and postsynaptic involvement in certain cases (Kullmann and Nicoll, 1992; Sjöström et al., 2007; Loebel et al., 2013; Costa et al., 2015). Generally, the locus of expression depends on factors such as animal age, induction protocol, and synapse type (Isaac et al., 1997; Corlew et al., 2007; Larsen and Sjöström, 2015). Indeed, there appears to be tremendous diversity in the cellular mechanisms that contribute to the expression of long-term potentiation (LTP) and depression (LTD) at central synapses (Malenka and Bear, 2004; Sjöström et al., 2008; Castillo, 2012). This diversity likely helps ensure the proper functioning of information storage by way of redundancy (Malenka and Bear, 2004; Murphy and Corbett, 2009). Despite this overwhelming diversity, the functional consequences of the locus of expression are actually quite poorly understood. Only a handful of recent theoretical studies show computational benefits from pre- and postsynaptic expression, such as memory savings and improved receptive field discriminability (Costa et al., 2015, 2017).

The locus of expression may thus hold distinct implications for neural coding and is therefore an important variable to resolve. For example, by modifying release probability, presynaptic expression not only affects the synaptic weight but also the reliability (Otmakhov et al., 1993) and short-term synaptic dynamics of neurotransmission (Markram and Tsodyks, 1996; Sjöström et al., 2007). Synaptic dynamics, such as short-term facilitation or depression, describe changes in synaptic strength that occur over the course of milliseconds to minutes (Zucker and Regehr, 2002; Abbott and Regehr, 2004; Fujisawa et al., 2008; Regehr, 2012). Such changes of synaptic efficacy have been proposed to underpin functionalities such as promotion of stability (Seeholzer et al., 2019), adaptation (Chance et al., 1998), decorrelation and burst detection (Lisman, 1997; Goldman et al., 2002), dynamic gain control (Abbott et al., 1997), detection of temporal coherence (Tsodyks and Markram, 1997), and working memory (Fujisawa et al., 2008; Costa et al., 2017). Postsynaptic expression, on the other hand, typically changes only the gain of synaptic transmission (Pananceau et al., 1998; Selig et al., 1999) (although see Poncer and Malinow, 2001), which in turn may affect signal to noise (Otmakhov et al., 1993). Whether long-term plasticity alters short-term plasticity thus has important computational implications. The locus of plasticity expression therefore matters.

The primary source of noise in synaptic transmission is derived from the probabilistic nature of neurotransmitter release (Otmakhov et al., 1993; Costa et al., 2017). As the coefficient of variation (CV) serves as a handy metric of noise due to synaptic release, changes in the CV due to e.g., the induction of long-term plasticity therefore imply presynaptic expression of plasticity (Bekkers and Stevens, 1990; Malinow and Tsien, 1990; Faber and Korn, 1991; Costa et al., 2017). Using CV analysis, it is therefore possible to resolve the locus of plasticity expression at central synapses. Here we provide basic instructions for how to carry out CV analysis, including tips and tricks for circumventing shortcomings and avoiding pitfalls.

## Materials and Equipment

### Animals and Ethics Statement

The animal study was reviewed and approved by the Montreal General Hospital Facility Animal Care Committee (The MGH FACC), and adhered to the guidelines of the Canadian Council on Animal Care (CCAC). P11-16 C57BL/6J mice were anesthetized with isoflurane and sacrificed once the hind-limb withdrawal reflex was lost. Every attempt was made to ensure minimum discomfort to the animals.

### Acute Slice Electrophysiology

After decapitation, the brain was removed and placed in ice-cold (∼4°C) artificial cerebrospinal fluid (ACSF), containing in mM: 125 NaCl, 2.5 KCl, 1 MgCl_2_, 1.25 NaH_2_PO_4_, 2 CaCl_2_, 26 NaHCO_3_, and 25 glucose, bubbled with 95% O_2_/5% CO_2_. Osmolarity of the ACSF was adjusted to 338 mOsm with glucose. Oblique coronal 300-μm-thick acute brain slices were prepared using a Campden Instruments 5000 mz^–2^ vibratome (Lafayette Instrument, Lafayette, IN, United States). Brain slices were kept at ∼33°C in oxygenated ACSF for ∼15 min and then allowed to cool at room temperature for at least one hour before we started the recordings. We carried out experiments with ACSF heated to 32–34°C with a resistive inline heater (Scientifica Ltd.), with temperature recorded and verified offline. Recordings were truncated or not used if outside this range.

We patched neurons with pipettes (4–6 MΩ) pulled from medium-wall capillaries using a P-1000 electrode puller (Sutter Instruments, Novato, CA, United States), and filled with a gluconate-based current-clamp solution containing (in mM): 5 KCl, 115 K-gluconate, 10 K-HEPES, 4 Mg-ATP, 0.3 Na-GTP, 10 Na_2_-phosphocreatine, adjusted to pH 7.2–7.4 with KOH. For 2-photon microscopy (see below), internal solution was supplemented with 10 μM Alexa Fluor 594 (Life Technologies, Carlsbad, CA, United States). Osmolarity of internal solution was adjusted to 310 mOsm with sucrose (Abrahamsson et al., 2016; Lalanne et al., 2016). Whole-cell recordings were amplified with BVC-700A amplifiers (Dagan Corporation, Minneapolis, MN, United States) or Multiclamp 700B amplifiers (Molecular Devices, San Jose, CA, United States). Voltage signals were first filtered at 5 kHz and then digitized at 10 kHz using PCI-6229 boards (National Instruments, Austin, TX, United States) controlled by custom software (Sjöström et al., 2001) running in Igor Pro 8 (Wavemetrics Inc., Lake Oswego, OR, United States) on a SuperLogics (Natick, MA, United States) computer.

Neurons were patched at 400× magnification with infrared video Dodt contrast (built in-house from Thorlabs parts) on a custom-modified SliceScope microscope (Scientifica Ltd., Uckfield, United Kingdom) (Abrahamsson et al., 2017). Primary visual cortex was identified by the presence of layer 4. Layer-5 (L5) pyramidal cells (PCs) were then targeted based on their large somata, thick apical dendrites, and distinctive triangular shape. We verified cell morphology using 2-photon microscopy (Figures 2A,B, 5Dii).

To compensate for their sparse connectivity (Song et al., 2005; Abrahamsson et al., 2017), connected L5 PC pairs were targeted for recording by the quadruple whole-cell recording approach, enabling us to test for 12 possible connections simultaneously (Abrahamsson et al., 2016; Lalanne et al., 2016). Seals were formed with four cells and then quickly and successively broken through to avoid plasticity washout. To find connections, we evoked in each cell five spikes at 30 Hz by current injections (5 ms duration; 1.3 nA amplitude) every 20 s for 10–20 repetitions. Spikes in different cells were separated by >700 ms to ensure that long-term plasticity was not accidentally induced (Sjöström et al., 2003; Lalanne et al., 2016). If no EPSPs were found, all four recordings were interrupted, and another four nearby cells were patched with fresh pipettes. If at least one sufficiently large connection was found (>∼0.3 mV, to ensure good signal-to-noise ratio), the baseline of the experiment was started. Perfusion temperature, input resistance, resting membrane potential or holding current, and EPSP amplitude were continuously monitored online and reassessed offline. Series resistance was not compensated. Liquid junction potential (10 mV) was not accounted for. As quality selection criteria, we required that input resistance change less than 30% and resting membrane potential less than 8 mV over the time course of the experiment, and that baseline period was stable as measured with a *t*-test of Pearson’s *r* (Sjöström et al., 2003, 2007; Buchanan et al., 2012; Abrahamsson et al., 2017). If these measures were stable over a 15-min-long period, LTD or LTP was elicited by repeated pre- and postsynaptic spike pairings. The LTD induction consisted on five spikes evoked in both pre- and postsynaptic cells at 20 Hz, repeated 15 times every 10 s, displaced by Δ*t* = 25ms pre- relative to postsynaptic spike. Similarly, LTP induction consisted of five spikes evoked in pre- and postsynaptic cells at 50 Hz, repeated 15 times every 10 s, displaced by Δ*t* = 10ms. In the post-pairing period, the spike bursts were continued up to 180 repetitions, for a total of 75 min.

### Two-Photon Laser-Scanning Microscopy

Two-photon laser-scanning microscopy was performed with an imaging workstation custom-built from a SliceScope (Scientifica Ltd., United Kingdom) microscope (Buchanan et al., 2012). Detectors were Scientifica 2PIMS-2000 or custom-built based on R3896 bialkali photomultipliers (Hamamatsu, Bridgewater, NJ, United States) and scanners were 6215H 3-mm galvanometric mirrors (Cambridge Technology, Bedford, MA, United States). Two-photon excitation was achieved using a MaiTai HP (Spectraphysics, Santa Clara, CA, United States) titanium-sapphire laser tuned to 820 nm to excite Alexa Fluor 594 fluorescence. Lasers were gated with SH05/SC10 (Thorlabs) shutters, and manually attenuated with a polarizing beam splitter in combination with a half-lambda plate (Thorlabs GL10-B and AHWP05M-980). Laser output was monitored with a power meter (Newport 1916-R with 818-SL). Fluorescence was collected with Semrock (FF665, FF01-680/SP-25) and Chroma filters (t565lpxr, ET630/75m, ET525/50m). Laser-scanning Dodt contrast was achieved by collecting the laser light after the spatial filter with an amplified diode (Thorlabs PDA100A-EC). Imaging data were acquired using customized variants of ScanImage version 3.7 (Pologruto et al., 2003) running in MATLAB (The MathWorks, Natick, MA, United States) via PCI-6110 boards (National Instruments).

After each whole-cell recording, L5 PC morphologies were acquired as stacks of 512-by-512-pixel slices (∼1.5 pixels/μm), with each slice spaced by 1 μm. Each slice was an average of 3 red-channel frames acquired at 2 ms per line. Morphologies shown (Figures 2A,B, 5Dii) are pseudo-colored maximum-intensity projections of such 3D stacks compiled with ImageJ (NIH, United States).

### Statistics

Unless otherwise noted, results are reported as the mean ± standard error of the mean (SEM). Significance levels are denoted using asterisks (^∗^*p* < 0.05, ^∗∗^*p* < 0.01, ^∗∗∗^*p* < 0.001). All pairwise comparisons were carried out using a two-tailed Student’s *t*-test for equal means. If an equality of variances *F* test gave *p* < 0.05, we employed the unequal variances *t*-test. Wilcoxon–Mann–Whitney’s non-parametric test was always used in parallel to the *t*-test, with similar outcome. Statistical tests were performed in Igor Pro (Wavemetrics Inc.).

### Simulations

Coefficient of variation analysis simulations were Monte-Carlo based with 150 repetitions of individual long-term plasticity experiments, carried out in Igor Pro. Each experiment was simulated with a baseline period consisting of 60 responses and a post-induction baseline of 240 responses. In real life, this would correspond to a 10-min baseline with an inter-stimulus interval of 10 s, followed by a 40-min-long post-pairing baseline, which is representative of our actual experiments (Sjöström et al., 2001, 2003; Abrahamsson et al., 2017). The number of release sites was fixed to *n* = 5, which is representative of a typical L5 PC-to-PC monosynaptic connection (Markram et al., 1997). To illustrate presynaptically expressed LTD (Sjöström et al., 2003, 2007), the probability of release was initially set to *p*_release_ = 0.55, and was reduced to *p*_release_ = 0.4 after the induction (which are representative values, e.g., see Costa et al., 2015), while the quantal amplitude was fixed at q = 0.35 mV.

Individual responses were simulated by drawing from a binomial distribution. Noise due to background activity, the amplifier, etc., was drawn from a zero-mean normal distribution with a standard deviation of 0.1 mV, which is representative of our experiments. Background noise was fixed and did not change throughout the simulated experiments.

For the outlier simulations, a single response in the baseline period was systematically increased by adding 0.1*e*^−3^×2^j^mV, where j = {0,…,5}, of which three steps are shown in Figures 4Ci–iii, with 150 simulation reruns for each step. The *z*-score, also known as the standard score, was calculated analytically from the binomial distribution parameters.

For the baseline trend simulations, a line with slope 0.6*e*^−6^×2^j^μV/min and zero mean was added to the simulated baseline responses, where j = {0,…,5}. As an illustration, three slopes are shown in Figures 5Ci–iii, each with 150 simulation reruns.

A simplified, accessible version of the simulation code is possible to download in Igor Procedure File format from GitHub: https://github.com/pj-sjostrom/Sim_CV_analysis. This code was created with a minimum number of user-modifiable parameters, to be pedagogical and relatively easy to experiment with for somebody who is new to the concept of CV analysis. This code includes the LTD simulations shown in Figures 4, 5, but extends to other scenarios, including LTP.

## The Quantal Theory of Synaptic Release

### Synaptic Release Is Quantized

Even in the absence of nervous impulses, single neurotransmitter-containing vesicles spontaneously fuse with specialized release sites in the presynaptic terminal, releasing their contents into the synaptic cleft through exocytosis (Südhof, 2013). In result, miniature postsynaptic potentials are generated, which represent the postsynaptic response due to the neurotransmitter contained in one vesicle (Fatt and Katz, 1952). This is the smallest unit of neurotransmission, which is known as a “quantum” (Fatt and Katz, 1952; Del Castillo and Katz, 1954). A synaptic bouton may contain multiple active zones or release sites (Korn et al., 1987; Korn and Faber, 1991; Maass and Zador, 1999), each of which are capable of probabilistically secreting a single quantum of neurotransmitter in response to an action potential (Isaacson and Walmsley, 1995; Korn and Faber, 1998; Maass and Zador, 1999). Although the release of multiple quanta has been documented many times (Tong and Jahr, 1994; Auger et al., 1998; Oertner et al., 2002; Lisman, 2009; Jensen et al., 2019), evoked responses are typically assumed to be due to the linear summation of single quanta released across multiple sites. Release at single sites has thus long been thought to be uniquantal as opposed to multiquantal (Lisman and Harris, 1993), even though recent studies suggest otherwise (Jensen et al., 2019). This assumption is central to the use of the binomial release model in CV analysis (see below and Box 1).

**Box 1. Assumptions underlying the binomial release model.**

Using the binomial distribution as a model of neurotransmitter release implies that several key assumptions were made. Here, we highlight several of these assumptions.

1. The release probability, *p*, of one quantal unit is uniform across all *n* release sites (Johnson and Wernig, 1971; McLachlan, 1978; Redman, 1990; Faber and Korn, 1991; Quastel, 1997). There is some evidence that this is in fact the case, for e.g., in the neocortex (Koester and Johnston, 2005) and hippocampus (Branco et al., 2008) (although see Walmsley et al., 1988).
2. The quantal size, *q*, is uniform across all *n* release sites and over a given epoch (McLachlan, 1978; Korn et al., 1987; Redman, 1990; Faber and Korn, 1991; Quastel, 1997). This requirement seems less biologically plausible. For example, because synaptic contacts are distributed in the dendritic arbor (Markram et al., 1997), dendritic cable filtering (Sjöström et al., 2008; Maheux et al., 2016) would likely ensure that the quantal size, *q*, varies from release site to release site. Although there is some evidence for mechanisms normalizing synaptic weights across the dendritic arbor (Magee, 2000; Magee and Cook, 2000; Häusser, 2001), there is also evidence to the contrary (Williams and Stuart, 2002; Nevian et al., 2007).
3. Each of the *n* release sites may secrete at most one quantum per action potential (Triller and Korn, 1982; Korn et al., 1987; Korn and Faber, 1991; Quastel, 1997), which is known as the “one vesicle hypothesis” (Korn and Faber, 1991; Quastel, 1997) (although see Tong and Jahr, 1994; Auger et al., 1998; Oertner et al., 2002; Lisman, 2009; Jensen et al., 2019). Considering that the neurotransmitter contents of one quantum is likely sufficient to saturate postsynaptic receptors (Redman, 1990; Lisman and Harris, 1993), it follows that—to satisfy the requirement for linear summation—uniquantal release from central synapses is thought to occur across multiple, spatially segregated release sites (Lisman and Harris, 1993).
4. Release is independent across all *n* sites (Johnson and Wernig, 1971; McLachlan, 1978; Quastel, 1997). This implies that there is no interaction or correlation of release events across adjacent sites and that released quanta summate linearly (Quastel, 1997).
5. The number of *n* release sites remains constant. This is probably true for early LTP in many cases, although new synaptic contacts are likely to be formed in late LTP (2–3 h after induction) (Geinisman et al., 1993; Bolshakov et al., 1997; Korn and Faber, 1998; Loebel et al., 2013). But *n* can also be affected by so-called “AMPAfication” of silent NMDA-only synapses, which occurs in very early development (Isaac et al., 1995, 1996; Liao et al., 1995; Kerchner and Nicoll, 2008)

Whether or not all five points hold true for all synapses is thus not always clear. As an example, the majority of Schaefer collateral inputs to hippocampal CA1 PCs are thought to feature a single active zone, yet multi-vesicular release has been suggested at these connections (Tong and Jahr, 1994; Oertner et al., 2002; Jensen et al., 2019). Either multiple vesicles can be released from one release site, or each active zone hosts multiple release sites. Either way, both points 3 and 4 above may thus be violated, calling into question the validity of the binomial release model. Having said that, the CV analysis method may still work, even if e.g., a Poisson rather than a binomial model of release should be employed (Korn and Faber, 1998), it is just that the analytical treatment becomes considerably more complex if e.g., multivesicular release occurs. It is furthermore possible to test experimentally for uni-vesicular versus multi-vesicular release (Saviane and Silver, 2007).

### Stochastic Release Is a Useful Source of Noise

The stochastic properties of neurotransmitter release result in fluctuations of the postsynaptic response (Otmakhov et al., 1993; Neher and Sakaba, 2003; Saviane and Silver, 2007), which are a prominent source of noise (Otmakhov et al., 1993; Neher and Sakaba, 2003). In contrast to experimental noise, which an investigator aims to reduce (Neher and Sakaba, 2003), the pattern of response noise fluctuations recorded from a neuronal connection provides insight into the molecular regulation of synaptic transmission (Katz and Miledi, 1972; Neher and Sakaba, 2003). This response noise is examined as part of fluctuation and quantal analysis to determine parameters governing synaptic efficacy (Scheuss and Neher, 2001) and has long been used for determining the pre- versus postsynaptic site of modification (Bekkers and Stevens, 1990; Bliss, 1990; Redman, 1990).

Another source of noise are membrane potential fluctuations produced by e.g., release from other synapses. As opposed to the experimental noise, this source of noise is intrinsic to the cell and cannot be reduced. It is possible, however, to subtract both these sources of background noise (see below) (Faber and Korn, 1991).

### Quantal Theory

The quantal theory of neurotransmitter release and the notion of a “quantum” was first described by Del Castillo and Katz (1954) at the neuromuscular junction in order to describe parameters influencing synaptic function and efficacy. Through their seminal recordings of the amphibian neuromuscular junction, it was observed that evoked potentials in a muscle fiber randomly fluctuate between integer multiples of the spontaneous miniature potential or basic quantal unit, *q* (Del Castillo and Katz, 1954; Korn and Faber, 1991, 1998). This finding has since been replicated at other synapse types (Redman and Walmsley, 1983; Korn et al., 1987; Isaacson and Walmsley, 1995). Quantal analysis relies on the pattern of fluctuations in evoked responses to calculate presynaptic factors influencing neurotransmitter release and postsynaptic factors influencing synaptic responsiveness, thereby allowing the locus of plasticity expression to be determined (Malinow and Tsien, 1990; Redman, 1990; Isaac et al., 1996; Reid and Clements, 1999; Enoki et al., 2009).

### Quantal Analysis Relies on Response Fluctuations

In quantal statistical models of neurotransmitter release, the mean synaptic response, μ, and its variance, σ^2^, depend on: (1) the probability that one quantum will be released, *p*, from the readily releasable pool of vesicles at the nerve terminal; (2) the total number of active release sites, *n*; and (3) the amplitude of the synaptic response produced by one quantum, *q* (Del Castillo and Katz, 1954; Martin, 1966; Korn et al., 1986). If a binomial distribution of responses is assumed (Box 1), the mean and variance are the expected value, *E*[*X*], and the variance, *Var*[*X*], of the response statistic *X*:

(1) E⁢[X]=μ=n⁢p⁢q

(2) $$V\,a\,r\,\left[X\right]=\sigma^{2}=n\,p\,\left(1-p\right)\,q^{2}$$

In this view, the parameter *n* corresponds to the number of active zones (Triller and Korn, 1982; Faber and Korn, 1991) or independent functional release sites (Bekkers and Stevens, 1990; Bliss, 1990; Korn and Faber, 1991). However, some debate still remains surrounding this definition (Scheuss and Neher, 2001). For example, *n* has alternatively been proposed to represent the maximum number of quanta available for evoked release at a given synapse (Redman, 1990; Isaacson and Walmsley, 1995), i.e., the number of docked vesicles or the size of the readily releasable pool (Kaeser and Regehr, 2017). Here, we are adhering to the more common view that *n* corresponds to the number of release sites.

### Changes in *p* and *q* Reveal the Locus of Expression

Presynaptic expression of plasticity is mediated by changes to the properties of vesicular release, typically the probability of release, *p* (Bekkers and Stevens, 1990; Chen and Regehr, 1997; Enoki et al., 2009) (reviewed by Castillo, 2012). Classically, the number of active release sites, *n*, was also considered to be a presynaptic parameter (Bekkers and Stevens, 1990; Faber and Korn, 1991). However, *n* has also been shown to be affected by postsynaptic events such as the unsilencing of AMPA receptors, which occurs more commonly in early development (Isaac et al., 1995, 1996; Liao et al., 1995; Kerchner and Nicoll, 2008). Furthermore, changes in *n* likely occur during the protein synthesis-dependent phase of late LTP (Geinisman et al., 1993; Bolshakov et al., 1997; Korn and Faber, 1998; Loebel et al., 2013). Here, we consider *n* to be stable for the duration of our experiments (∼1 h; Box 1). A presynaptic locus is then assumed to be mediated by changes in *p* (Box 2).

**Box 2. What is the point of using 1/CV^2^ instead of CV?**

It may seem counterintuitive and unnecessarily cumbersome to plot 1/CV^2^, normalized, versus the normalized amplitude in CV analysis. The rationale for this practice stems from the fact that—if you assume a binomial model of vesicular release—the probability of release, *p*, is proportional to 1/CV^2^. In other words, you can to a first approximation read off the change in release probability, *p*, from the *y*-axis when the CV analysis is represented in this manner, which is a handy advantage.

To show that *p*∝1/CV^2^, we combine the expected value *E*[*X*] (Eq. 1) and the variance *Var*[*X*] of the binomial distribution (Eq. 2), and plug these into the expression for the CV, which is the standard deviation over the mean.

$$\left\{ \begin{array}{c} \text{CV}=\frac{\sigma }{\mu }\\ E\,\left[X\right]=n\,p\,q=\mu \\ V\,a\,r\,\left[X\right]=n\,p\,\left(1-p\right)\,q^{2}=\sigma^{2} \end{array}\right.$$

$$\Rightarrow {\text{CV}}^{2}={\left(\frac{\sigma }{\mu }\right)}^{2}=\frac{n\,p\,\left(1-p\right)}{{\left(n\,p\right)}^{2}}=\frac{1-p}{n\,p}$$

Here, the scaling resulting from the quantal amplitude, *q*, vanishes. Solving for *p* gives:

$$p=\frac{1}{n\,{\text{CV}}^{2}+1}$$

So, if we assume that the number of release sites, *n*, does not change after the induction of plasticity, it follows that:

$$∴p\propto \frac{1}{{\text{CV}}^{2}}$$

Although the number of release sites, *n*, may change in late LTP by growth of new synaptic connections (Geinisman et al., 1993; Bolshakov et al., 1997; Korn and Faber, 1998; Loebel et al., 2013), it is reasonable to assume that *n* does not change in early LTP (Box 1). This assumption, however, is a key caveat of assuming the binomial distribution in CV analysis.

**What is special about the diagonal?**

**FIGURE 1:**
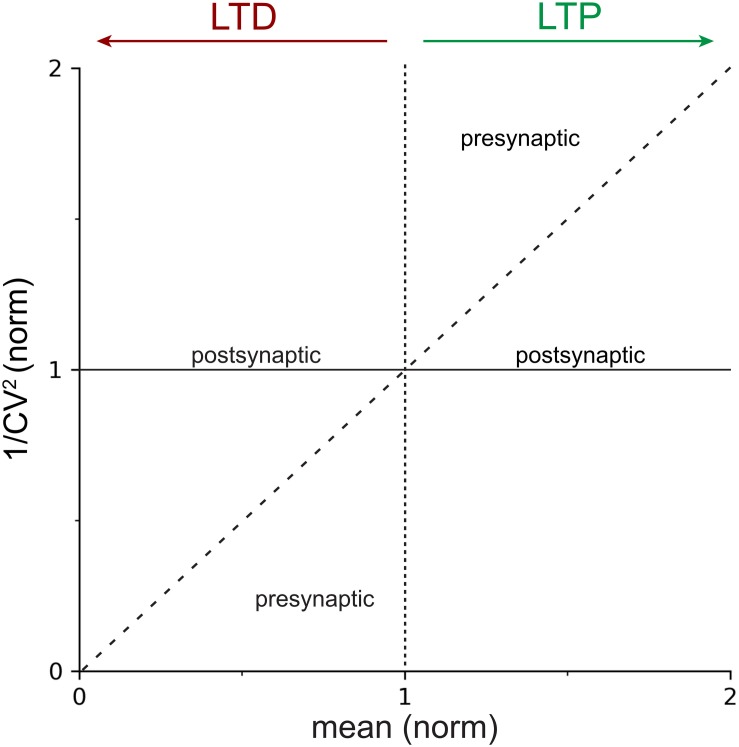
Locus of expression areas in the CV analysis plot. Normalized 1/CV^2^, a proxy for the probability of release *p* (Box 2), is plotted against the normalized mean μ(norm), which is a measure of synaptic strength. The solid horizontal line at *y=1* indicates 100% 1/CV^2^(norm), or no change in *p*. The dotted vertical line at *x=1* delineates LTP (to the right) from LTD (to the left). The dashed diagonal line with slope Δ*y*/Δ*x* = 1 demarcates presynaptic from postsynaptic expression of plasticity (Box 2). In other words, data that falls on or close to the continuous horizontal line should be considered to be postsynaptically expressed, whereas data that is above the dashed diagonal for LTP, or below it for LTD, should be considered presynaptically expressed. Mixtures of pre- and postsynaptic expression is also possible (Sjöström et al., 2007), which results in data points scattered between the dashed diagonal and the continuous horizontal lines.

One additional advantage of plotting 1/CV^2^ versus the mean is that the diagonal line, Δ*y*/Δ*x* = 1, can be used as a demarcation line to determine whether expression is pre- or postsynaptic (Figure 1). To show this, we again use the expressions for the expected value, *E*[*X*], and the variance, *Var*[*X*], of the binomial distribution (Eqs 1 and 2), and combine these with the expression for the CV.

$$\left\{ \begin{array}{c} \text{CV}=\frac{\sigma }{\mu }\\ E\,\left[X\right]=n\,p\,q=\mu \\ V\,a\,r\,\left[X\right]=n\,p\,\left(1-p\right)\,q^{2}=\sigma^{2} \end{array}\right.$$

$$\Rightarrow {\text{CV}}^{2}={\left(\frac{\sigma }{\mu }\right)}^{2}=\frac{n\,p\,\left(1-p\right)}{{\left(n\,p\right)}^{2}}=\frac{1-p}{n\,p}$$

We solve for 1/CV^2^ and normalize with respect to the initial probability of release, *p*_0_. We also define a presynaptic change in synaptic strength due to altering the probability of release, *c*_pre_ = *p*/*p*_0_, to explore what happens when expression is only presynaptic. In this scenario, the *y* coordinate in the CV analysis plot is:

$${\frac{1}{{\text{CV}}^{2}}}_{\text{norm}}=\frac{p}{1-p}\,\frac{1-p_{0}}{p_{0}}=\frac{c_{\text{pre}}\,\left(1-p_{0}\right)}{1-c_{\text{pre}}\,p_{0}}$$

Here, it is useful to note that this above expression does not depend on the quantal amplitude, *q*. Similarly, the *x* coordinate, μ_*norm*_, in the CV analysis plot is:

$$\mu_{\text{norm}}=\frac{n\,p\,q}{n_{0}\,p_{0}\,q_{0}}=c_{\text{pre}}\,c_{\text{post}}$$

where

$$\left\{ \begin{array}{c} n=n_{0}\\ c_{\text{pre}}=\frac{p}{p_{0}}\\ c_{\text{post}}=\frac{q}{q_{0}} \end{array}\right.$$

As before, we assume that the number of release sites, *n*, remains unaltered. In the scenario where plasticity is solely presynaptic, *c*_*post*_ reduces to 1, so we are left with μ_norm_ = *c*_pre_. Here, the end coordinate becomes:

$$\left(c_{\text{pre}},\frac{c_{\text{pre}}\,\left(1-p_{0}\right)}{1-c_{\text{pre}}\,p_{0}}\right)$$

Therefore, the slope of an imagined line from the starting coordinate (1,1) to this end point is:

$$\frac{\Delta \,y}{\Delta \,x}=\frac{\frac{c_{\text{pre}}\,\left(1-p_{0}\right)}{1-c_{\text{pre}}\,p_{0}}-1}{c_{\text{pre}}-1}=\frac{1}{1-c_{\text{pre}}\,p_{0}}$$

Since both *c*_*pre*_ and *p*_0_ are positive, non-zero numbers, it follows that Δ*y*/Δ*x* > 1. Ergo, presynaptically expressed plasticity gives rise to data points above the unitary diagonal line Δ*y*/Δ*x* = 1 for LTP. In the case of LTD, the scenario is the inverse; presynaptically expressed plasticity gives rise to data points below the diagonal (Figure 1).

In the case where plasticity is solely postsynaptically expressed, we are left with μ_norm_ = *c*_post_, so the final CV coordinate is now:

$$\left(c_{\text{post}},\frac{c_{\text{pre}}\,\left(1-p_{0}\right)}{1-c_{\text{pre}}\,p_{0}}\right)=\left(c_{\text{post}},\frac{1-p_{0}}{1-p_{0}}\right)=\left(c_{\text{post}},1\right)$$

which implies a line parallel to the *x*-axis:

$$\frac{\Delta \,y}{\Delta \,x}=\frac{1-1}{c_{\text{post}}-1}=0$$

This finding is in effect trivial, since we already observed above that $1/{\text{CV}}_{\text{norm}}^{2}$ did not depend on the quantal amplitude, *q*. It is also consistent with the above observation that *p*∝1/CV^2^ since a line parallel to the *x*-axis implies that the probability of release *p* remains unaltered as the mean μ is increased or decreased.

In practice, since CV analysis relies on a finite number of data points in the baseline and post-induction period, the slope of the line between the coordinate (1, 1) and the end point will suffer from inaccuracy, due to the noise inherent in the stochasticity of release. This means pre- and postsynaptically expressed plasticity will not always give rise to data points on opposite sides of the diagonal demarcation line (e.g., see Figures 4 and 5), especially for experiments with baseline period with relatively few responses. Plasticity can of course also be expressed as a mixture of pre- and postsynaptic mechanisms (Sjöström et al., 2007; Costa et al., 2015), in which case data points may consistently end up on or close to the diagonal line.

Conversely, postsynaptic expression of plasticity is reflected as a change in the regulation, turnover, or responsiveness of postsynaptic receptors (Sheng and Kim, 2002; MacDougall and Fine, 2014; Costa et al., 2017). The quantal size, *q*, depends upon the number and properties of postsynaptic receptors activated by a quantum as well as by the amount of transmitter contained in one vesicle (Korn and Faber, 1998). Although *q* may thus in principle be influenced by both pre- and postsynaptic factors, the quantal size is commonly assumed to relate to postsynaptic mechanisms. In other words, vesicle size and transmitter loading are assumed to be both stereotyped and not plastic (Bliss, 1990; Faber and Korn, 1991; Korn and Faber, 1998).

### Statistical Models Are Used to Estimate Synaptic Parameters

According to Del Castillo and Katz (1954) and many others (Johnson and Wernig, 1971; McLachlan, 1978; Korn et al., 1987; Bekkers and Stevens, 1990; Redman, 1990), the frequency distribution of evoked postsynaptic responses due to probabilistic presynaptic release follows binomial statistics. Poisson statistics may be more realistic in certain cases, for example in low Ca^2+^-to-Mg^2+^ conditions when *p* is very low (Del Castillo and Katz, 1954; Martin, 1966). However, binomial statistics are assumed in the majority of studies of release.

The choice of release statistics comes with inherent assumptions. When the binomial model is relied upon, it is for example implicitly assumed that the release probability, *p*, and quantal size, *q*, are uniform across all *n* release sites (Box 1). These assumptions have the added benefit of simplifying the relationships between the synaptic parameters *n*, *p*, and *q* (McLachlan, 1978; Redman, 1990; Faber and Korn, 1991; Korn and Faber, 1991; Costa et al., 2017). Another useful consequence is the binomial model provides a simple theoretical framework for identifying the locus of expression of long-term plasticity by analysis of the CV (Box 2; Bekkers and Stevens, 1990; Malinow and Tsien, 1990; Faber and Korn, 1991). However, even if the constraints for the binomial release model are not met, CV analysis may still work adequately (Box 1; Faber and Korn, 1991).

## Principles of Cv Analysis

### The Basis for CV Analysis in Intuitive Terms

In probability theory and statistics, the CV—which is defined as the standard deviation σ divided by the mean μ—is a general standardized measure of dispersion of a probability or frequency distribution. The CV is, in other words, an experimentally useful measure of noise, or normalized overall variability (Abdi, 2010). For this reason, the CV is also known as the relative standard deviation. Since the majority of the noise at a synapse is due to the stochastic nature of quantal neurotransmitter release (Otmakhov et al., 1993; Costa et al., 2017), changes in noise as indicated by alterations in the CV are useful, since such changes suggest a presynaptic locus of that change, i.e., due to a change in *p* (Bekkers and Stevens, 1990; Malinow and Tsien, 1990; Faber and Korn, 1991). Conversely, an on-average alteration in synaptic strength without a concomitant change in the CV would by the same line of reasoning appear to be due to postsynaptic changes in *q*, e.g., by regulation of AMPA receptors (Kauer et al., 1988; Isaac et al., 1995; Liao et al., 1995; Barria et al., 1997; Nicoll and Malenka, 1999). This latter observation, however, assumes that vesicle neurotransmitter loading is fixed and stereotyped (Box 1). In summary, an overall intuitive understanding of CV analysis should thus be based on the observation that changes in synaptic noise are primarily due to presynaptic expression. Conversely, no changes in noise during long-term plasticity suggests that expression is postsynaptic.

### Binomial Release Statistics

In the context of neurotransmission, the CV is represented by the standard deviation, σ, of a set of evoked synaptic responses divided by their mean, μ, taken over a given time period (Bekkers and Stevens, 1990; Malinow and Tsien, 1990; Faber and Korn, 1991; Costa et al., 2017):

(3) $$\text{CV}=\frac{\sigma }{\mu }$$

To extract specific synaptic release parameters, it is useful to apply a specific statistical model. A typical choice is the binomial release model (Box 1), although it is important to understand that the CV is a general measure of noise and that the CV is not in and of itself linked to any particular statistical model.

In terms of binomial statistics, μ is the mean synaptic efficacy given by the expected value of the random variable *X*, which is *E*[*X*] = μ = *npq* (Eq. 1), and the standard deviation is derived from the variance (Eq. 2) as $\sigma =\sqrt{V\,a\,r\,\left[X\right]}=\sqrt{n\,p\,\left(1-p\right)\,q^{2}}$. These mathematical relationships have been described many times in greater detail in the previous literature, and we refer the reader to these papers for a more in-depth treatment (Johnson and Wernig, 1971; McLachlan, 1978; Faber and Korn, 1991).

Typically, 1/CV^2^ rather than CV is plotted in most studies (Figure 1). This perhaps counterintuitive practice can be explained by the fact that 1/CV^2^ is proportional to the probability of release (Box 2). This practice is furthermore justified by the observation that the unitary diagonal line in a 1/CV^2^ versus μ plot (Figure 1) is a handy demarcation line between pre- and postsynaptic expression (Box 2). In this context, it is worth noting that the analytical expression for the CV is independent of the quantal size, *q* (Bliss, 1990; Malinow and Tsien, 1990; Faber and Korn, 1991; Korn and Faber, 1991; Costa et al., 2017), as derived from Eqs 1–3:

$$\text{CV}=\left(\frac{\sigma }{\mu }\right)=\sqrt{\frac{1-p}{n\,p}}$$

This fact reflects the observation in the above intuitive introduction to CV analysis that postsynaptic changes should not affect synaptic noise levels. Again, this is because the variation at individual release sites predominately stems from the stochastic nature of neurotransmitter release (Otmakhov et al., 1993; Costa et al., 2017) and the CV is a metric of noise (Abdi, 2010; Costa et al., 2017).

To ensure that the CV reflects synaptic noise, characteristic of stochastic release, it has been argued that it should be corrected for the background noise (Faber and Korn, 1991):

$$\sigma^{2}=\sigma_{\text{measured}}^{2}-\sigma_{\text{background}}^{2}$$

In practice, we find that subtracting the background noise has little or no impact on the locus of expression, as long as the background noise is stable across recordings.

## Results

### Expected Outcomes

Coefficient of variation analysis compares the relative change of synaptic parameters before and after induction of plasticity (Faber and Korn, 1991), which in practical terms means we work with normalized values of CV and mean synaptic strength, μ. In the standard CV analysis plot (Figure 1), the normalized change in synaptic strength, μ(norm), thus indicates whether LTP or LTD took place, while appreciable changes in 1/CV^2^(norm) serve as a proxy for modifications in presynaptic release (Bekkers and Stevens, 1990; Malinow and Tsien, 1990; Faber and Korn, 1991; Costa et al., 2017), as outlined above. Whether a change in 1/CV^2^(norm) is appreciable or not is determined by comparing the outcome to the diagonal line (Figure 1 and Box 2; Sjöström et al., 2003, 2007; Buchanan et al., 2012; Abrahamsson et al., 2017).

If 1/CV^2^(norm) changes at least to the same degree as the mean synaptic efficacy, μnorm, this supports a presynaptic locus of plasticity expression (Figure 1). On the contrary, if 1/CV^2^(norm) remains relatively unaffected as the mean response μ(norm) changes, this is evidence to support a postsynaptic locus of plasticity expression (Figure 1; Korn and Faber, 1991; Reid and Clements, 1999). Of course, forms of plasticity may involve both pre- and postsynaptic modifications (Kullmann and Nicoll, 1992; Sjöström et al., 2007; Loebel et al., 2013; Costa et al., 2015).

**FIGURE 2:**
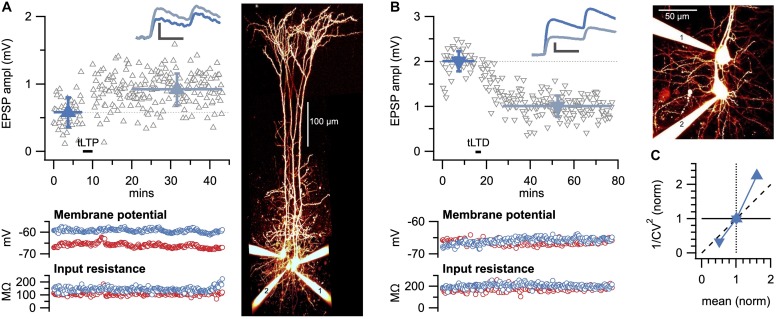
Sample LTP and LTD experiments show presynaptic expression. **(A)** Sample spike-timing-dependent plasticity experiment with Δ*t* = 10ms temporal difference between pre- and postsynaptic spike trains evoked at 50 Hz (Sjöström et al., 2001) for which LTP was evoked (EPSP before, dark blue: 0.58 ± 0.03 mV versus after, light blue: 0.92 ± 0.02 mV, *p* < 0.001). Inset: average EPSP traces showed a change in paired-pulse ratio suggesting presynaptic expression (Sjöström et al., 2007). Scale bars: 0.5 mV, 20 ms. Bottom: membrane potential and input resistance of pre- and postsynaptic PCs were stable (red and blue, respectively). Right: flattened 2-photon imaging stack of Alexa-594-filled cells verified PC identity, with pre- and postsynaptic PCs denoted by 1 and 2, respectively. **(B)** Sample spike-timing-dependent plasticity experiment with Δ*t* = −25ms temporal difference between pre- and postsynaptic spike trains evoked at 20 Hz (Sjöström et al., 2001, 2003) for which LTD was elicited (before: 2.0 ± 0.04 mV versus after: 1.0 ± 0.02 mV, *p* < 0.001. Inset: change in paired-pulse ratio suggested presynaptic expression (Sjöström et al., 2003, 2007). Scale bars: 0.5 mV, 20 ms. Bottom: membrane potential and input resistance of pre- and postsynaptic PCs were stable (red and blue, respectively). Right: pre- and postsynaptic PCs are indicated by 1 and 2, respectively. **(C)** Coefficient of variation analysis of LTP (right-side-up triangle) and LTD experiments (upside-down triangle) in **(A,B)** both indicated a presynaptic locus of expression, in keeping with prior findings (Markram and Tsodyks, 1996; Sjöström et al., 2003, 2007).

Overall, CV analysis provides an estimate of the locus of plasticity expression without having to resolve precise changes in *n*, *p*, or *q* (Costa et al., 2017). This is useful, because quantifying changes in *n*, *p*, or *q*—known as quantal analysis—is labor intensive and typically requires specific experimental conditions (Larkman et al., 1992, 1997a,b). Another approach for directly quantifying changes in *n*, *p*, or *q*, known as variance-mean analysis, requires sequential changes in cation composition (Clements and Silver, 2000; Clements, 2003). However, CV analysis can readily be performed following plasticity experiments without prior preparation (Figure 2A), but this relative simplicity comes at the cost of not knowing the precise changes in *n*, *p*, and *q*. The two sample paired-recording experiments show how both LTP (Figure 2A) and LTD (Figure 2B) at L5 PC-PC connections are presynaptically expressed according to CV analysis (Figure 2C), in agreement with our prior findings (Sjöström et al., 2003, 2007).

**FIGURE 3:**
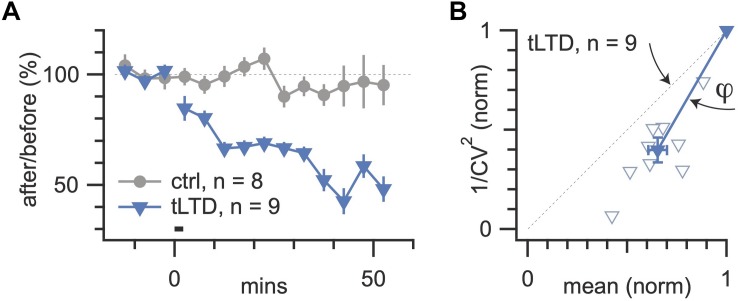
Neocortical LTD in L5 PCs is presynaptically expressed. **(A)** LTD expression at 20 Hz with Δ*t* = −25ms like in Figure 2B was robust across paired recordings, while no-induction controls were stable (LTD, blue triangles; 65 ± 5%, *n* = 9 versus control, gray circles; 97 ± 2%, *n* = 8, *p* < 0.001). **(B)** Coefficient of variation analysis consistently suggested a presynaptic locus of LTD expression, as all paired recordings gave rise to data points below the diagonal (angle φ = 16° ± 2°, *n* = 9, *p* < 0.001; see Figure 1).

To be able to draw robust conclusions about the locus of plasticity, it is essential to repeat across several long-term plasticity experiments (Figure 3). Here, the statistical significance of CV analysis can be assessed by comparing the angle φ of the outcome relative to the diagonal (Figure 3C and Box 2), as we have done before (Sjöström et al., 2003, 2007; Buchanan et al., 2012; Abrahamsson et al., 2017).

In summary, CV analysis is a straightforward method for estimating the locus of expression that can easily be implemented following a standard plasticity experiment without the need for any special preparations (Bekkers and Stevens, 1990; Malinow and Tsien, 1990; Sjöström et al., 2003, 2007). It is important, however, to be aware of the assumptions of the binomial distribution (Box 1) as well as several experimental pitfalls associated with CV analysis (see below).

### Caveats of CV Analysis

Like any other method, CV analysis comes with caveats (Faber and Korn, 1991; Korn and Faber, 1991; Costa et al., 2017). As a consequence, CV analysis may be misleading in some cases (Faber and Korn, 1991). Here, we show how to anticipate and circumvent some of the key shortcomings.

#### The Number of Activated Inputs Should Remain Constant

A constant number of afferents should be activated within and across trials (Redman, 1990; Faber and Korn, 1991; Saviane and Silver, 2007). Although it is possible to conduct CV analysis on synaptic responses evoked with extracellular stimulation (Bekkers and Stevens, 1990; Malinow and Tsien, 1990), there is with extracellular stimulation the potential for loss or gain of afferent fibers throughout the recording, which may complicate CV analysis by requiring corrections (Faber and Korn, 1991; Costa et al., 2017). This potential problem is not specific to CV analysis *per se*, but also applies to e.g., quantal analysis and variance-mean analysis. Furthermore, recordings that show evidence of polysynaptic connectivity violate the simple binomial model (McLachlan, 1978) and therefore complicate the interpretation of quantal parameters by precluding CV analysis (Faber and Korn, 1991; Korn and Faber, 1991; Costa et al., 2017) and require statistical adjustments (Faber and Korn, 1991; Reid and Clements, 1999).

Avoiding these problems can be achieved by interrogating monosynaptic connections using paired recordings (Korn and Faber, 1998; Saviane and Silver, 2007), which have been carried out e.g., in neocortex (Figure 2; Sjöström et al., 2003, 2007; Song et al., 2005; Lalanne et al., 2016) and hippocampus (Sayer et al., 1989; Bekkers and Stevens, 1990; Malinow, 1991; Debanne et al., 1999). However, identifying monosynaptic connections is technically challenging and time consuming, especially for synapse types with low connectivity rates. To alleviate this problem, multiple whole-cell recordings may be employed to increase the yield of identified monosynaptic connections (Figures 2, 3), as previously described by us (Sjöström et al., 2003, 2007; Song et al., 2005; Lalanne et al., 2016) and others (Perin et al., 2011; Perin and Markram, 2013; Peng et al., 2019).

It is also possible to circumvent the problem of accidental loss or gain of afferent inputs by using more direct optical methods such as 2-photon glutamate uncaging (Ellis-Davies, 2019; Mitchell et al., 2019) or optical quantal analysis (Oertner et al., 2002; Emptage et al., 2003; MacDougall and Fine, 2019; Padamsey et al., 2019). However, even with paired recordings or these more direct optical methods, it is still possible for the number of release sites *n* to change (Box 1).

#### Outlier Synaptic Responses Distort CV Analysis

The variation at single synaptic contacts—primarily driven by the stochastic and probabilistic nature of presynaptic release (Otmakhov et al., 1993; Costa et al., 2017)—significantly influences the overall observed variability i.e., fluctuations in evoked potentials between neuronal connections in the brain (Otmakhov et al., 1993; Crochet et al., 2005). This makes the CV an excellent proxy for presynaptic changes in release (Malinow and Tsien, 1990; Faber and Korn, 1991; Costa et al., 2017). However, it also indicates that the CV is sensitive to the variation and stability of synaptic parameters at each release site and is therefore vulnerable to measurement error in the presence of additional sources of variation (Faber and Korn, 1991; Korn and Faber, 1991).

**FIGURE 4:**
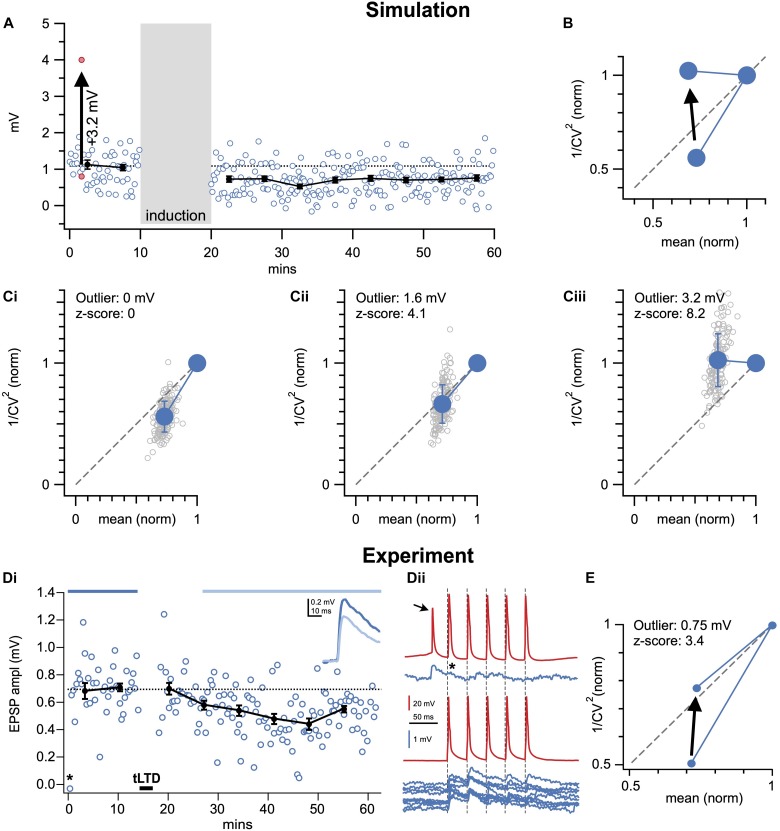
A single outlier response may corrupt CV analysis. **(A)** Sample Monte-Carlo simulation of an individual presynaptically expressed LTD experiment in which a single EPSP was shifted by 3.2 mV (*z*-score: 8.2) to produce a striking outlier (red dots). To enable comparison with experimental data (Figures 2, 3), the number of EPSPs, interstimulus intervals, background noise levels, amount of LTD, initial EPSP amplitude, et cetera were set to representative values (see section “Materials and Equipment”). **(B)** With a single outlier in the baseline period (*z*-score 8.2 as in **A**), CV analysis of LTD was on average biased to erroneously indicate post-instead of presynaptic expression (arrow). In the case of LTP, CV analysis would instead be biased toward presynaptic expression (not shown, but possible to simulate in downloadable code, see section “Materials and Equipment”), because the outlier would still artificially elevate the *y*-axis coordinate, just as for LTD. However, if the outlier is in the post-induction period, the bias is in the opposite direction. **(C)** As in **(A)**, 150 individual simulations (gray circles) were systematically repeated for single outliers of increasing *z*-score values (0, 4.1, and 8.2 shown in **Ci–iii**). The increasing outlier values systematically biased outcome toward a postsynaptic interpretation (summarized in **B**). **(D)** Sample LTD experiment (**Di**, Δ*t* = −25ms and 20 Hz as in Figures 2, 3) for which a spurious presynaptic spike (arrow, **Dii**, top red trace) resulted in undesirable short-term depression of subsequent EPSP (* in **Dii**, compare top to bottom blue sample traces), leading to an outlier EPSP in the time course (* in **Di**). **(E)** By including the outlier (* in **Di,ii**), CV analysis was biased toward postsynaptic interpretation (arrow). Here, this pitfall was avoided by removing the outlier (arrow starting point).

**FIGURE 5:**
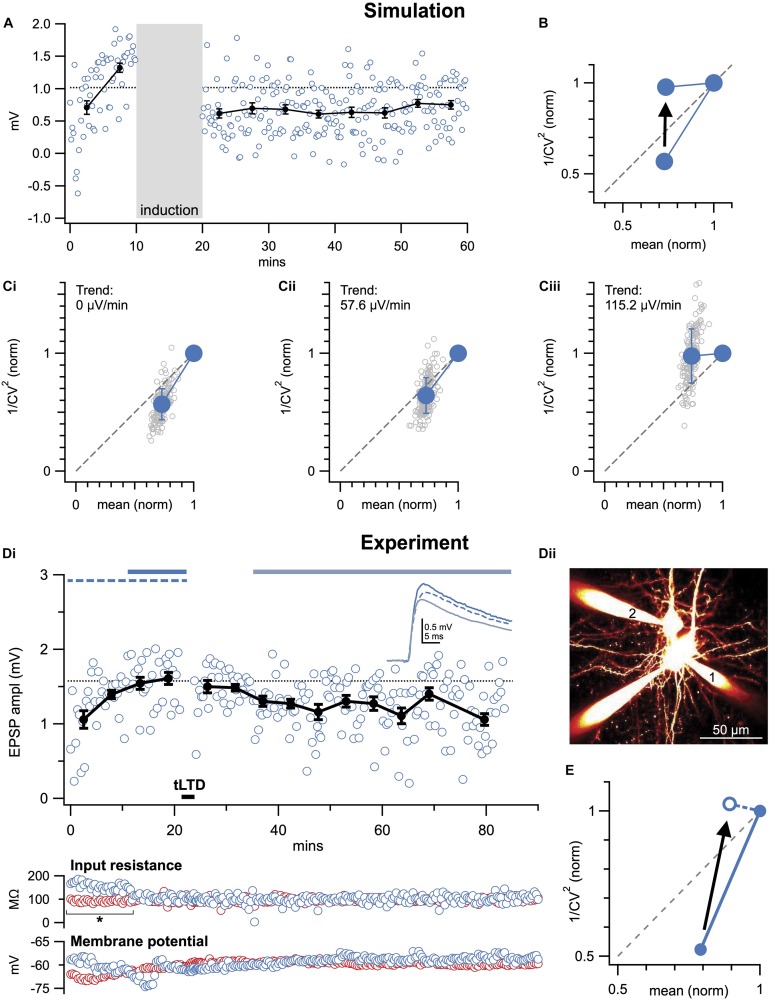
Baseline trends may corrupt CV analysis. **(A)** Sample Monte-Carlo simulation of an individual presynaptically expressed LTD experiment that was suffering from a strong baseline run-up (115.2 μV/min, see section “Materials and Equipment”). **(B)** With baseline trend (115.2 μV/min as in **A**), CV analysis was on average biased to erroneously indicate post-instead of presynaptic expression (arrow). In the case of LTP, CV analysis would instead be biased toward presynaptic expression (not shown, but possible to simulate in downloadable code, see section “Materials and Equipment”), because the baseline trend artificially elevates the *y*-axis coordinate. However, if the baseline trend is in the post-induction period, the bias is in the opposite direction. **(C)** As in **(A)**, 150 individual simulations (gray circles) were systematically repeated for different baseline trends (0, 57.6, and 115.2 μV/min shown in **Ci–iii**). The increasing baseline trend systematically biased outcome toward a postsynaptic interpretation (summarized in **B**). **(D)** Sample LTD experiment (**Di**, Δ*t* = −25ms and 20 Hz as in Figures 2, 3) at PC1 → PC2 connection **(Dii)** that suffered from an increasing baseline trend, coincident with a significant change in postsynaptic input resistance (bottom: blue circles, asterisk). Presynaptic input resistance and membrane potential are indicated in red. **(E)** By including the entire baseline period, CV analysis was biased toward postsynaptic interpretation (arrow). Here, this pitfall was avoided by removing the unstable baseline period, which was further supported by a significant change in input resistance (* in **Di**).

Extraneous sources of variation—for e.g., outliers due to stimulus failure or electrical artifacts (Oleskevich et al., 2000) and baseline trends and/or rundown (Reid and Clements, 1999)—significantly affect the CV and may mask the true locus of expression (Figures 4, 5). A straightforward solution to this caveat is to carefully inspect experiments for outlier responses and then individually exclude them from the CV analysis (Figures 4D,E). However, careful selection criteria for removing data points should be applied, otherwise bias will certainly be introduced. For example, electrical artifacts or spurious spiking (Figure 4D) are quite striking and useful selection criteria for identifying outliers. In other words, outliers should not be removed merely on the basis of being an outlier. Outliers should only be removed based on evidence for a cause of it being an outlier, such as spurious spiking (Figure 4D). Nevertheless, bias is a concern so removal of data points should be rare.

#### Unstable Baseline Distorts CV Analysis

Recordings should be evaluated for any trends resulting from rundown or instability, which may inflate estimates of the standard deviation, σ (McLachlan, 1978; Scheuss and Neher, 2001). The effect of baseline drift is illustrated in Figure 5; note that it is quite substantial even for relatively small baseline trends. Such trends can arise from gradual changes in cell input resistance, resting membrane potential, et cetera (Figure 5Di). It is therefore important to continuously monitor such parameters throughout long-term plasticity experiments (Figures 2A,B).

One solution to this problem is to systematically eliminate experiments above a threshold trend value, using a numerical selection criterion based on e.g., linear regression or bisection of the baseline period (Lalanne et al., 2016). By applying the same selection criteria to condition as well as control experiments (e.g., Figure 3), bias is avoided. We advise against detrending data, as it may introduce bias depending on the assumptions underlying the detrending algorithm. It is possible, however, to remove a portion of the baseline period that is unstable (Figure 5D), especially if doing so is supported by some independent selection criterion such as change in input resistance, resting membrane potential, or similar (Lalanne et al., 2016).

Gradual trends in variance or mean may also be addressed by binning 1/CV^2^ over time (Scheuss and Neher, 2001). For simplicity, we do not show this here, but we have relied on this approach before (Sjöström et al., 2003).

#### Alternative Interpretations Are Possible

Even under the best of circumstances, the results of CV analysis should be interpreted while considering the structure and function of the synapse type under investigation (Costa et al., 2017). To illustrate this point, consider NMDA-receptor-dependent LTP in hippocampal area CA1. Some studies have found that this form of plasticity is expressed as an increase in the probability of release, *p*, suggesting a presynaptic locus (Kullmann and Nicoll, 1992). However, this apparent change in the release probability may in fact be achieved postsynaptically by the conversion of silent to functional synapses (Glasgow et al., 2019). In this scenario, postsynaptic insertion of AMPA receptors may be erroneously interpreted as a presynaptic increase in the probability of release (Isaac et al., 1995, 1996; Liao et al., 1995; Kerchner and Nicoll, 2008). Synaptic unsilencing at the neuromuscular junction, on the other hand, is a mechanistically distinct presynaptic phenomenon (Wojtowicz et al., 1994).

In summary, alternative interpretations are often possible. This pitfall, however, is not limited to CV analysis as such but is a general caveat. Nevertheless, this means CV analysis should generally be supported by other methods for localizing the expression locus, such as analysis of failure rate (Malinow and Tsien, 1990; Faber and Korn, 1991), paired-pulse ratio (Figures 2A,B; Poncer and Malinow, 2001; Sjöström et al., 2007; Abrahamsson et al., 2017), NMDA:AMPA ratio (Watt et al., 2004; Sjöström et al., 2007), FM1-43 dye loading (Murthy et al., 1997; Zakharenko et al., 2001), spontaneous release (changes in frequency versus amplitude; Malgaroli and Tsien, 1992; Manabe et al., 1992; Abrahamsson et al., 2017), etc. Of these approaches, evaluating the paired-pulse ratio is likely the most straightforward option, as it can be readily performed in parallel with CV analysis, provided the experiments were carried out with paired pulses (Figures 2A,B). Since it relies on two responses rather than one as for CV analysis, paired-pulse ratio analysis is furthermore mathematically independent from CV analysis. Failure-rate and CV analyses, however, are essentially relying on the same theoretical framework and so are not independent methods, which means the corroborative power is limited. For further information regarding these techniques, we invite the reader to the review by Glasgow et al. (2019) in this research topic. Furthermore, modern techniques enable more direct measurements of locus of expression, e.g., using 2-photon glutamate uncaging (Ellis-Davies, 2019; Mitchell et al., 2019), optical glutamate sensors (Jensen et al., 2017, 2019; Durst et al., 2019), or optical quantal analysis (Oertner et al., 2002; Emptage et al., 2003; MacDougall and Fine, 2019; Padamsey et al., 2019). These more advanced methods may however require expensive specialized equipment.

## Discussion

We have provided a practical guide to using CV analysis for the purposes of investigating the locus of expression of long-term plasticity. We primarily directed this guide to beginners in the field, so we have tried to simplify key concepts to make them more accessible. We acknowledge that others have delved into the mathematical background with greater detail and rigor than we have here (McLachlan, 1978; Faber and Korn, 1991; Quastel, 1997); this was intentional.

Binomial statistics have been successfully applied to the study of quantal release at peripheral and central synapses for decades (Johnson and Wernig, 1971; McLachlan, 1978; Korn et al., 1987; Bekkers and Stevens, 1990; Malinow and Tsien, 1990). Nonetheless, the simplifying assumptions inherent in this model may not hold in all cases. Therefore, if resolving precise changes in synaptic parameters is required, it is possible to use alternative albeit more laborious approaches to accommodate potential non-uniformities in *p* and *q* (Silver et al., 1998; Reid and Clements, 1999; Saviane and Silver, 2007). However, it appears that the locus of plasticity expression can be reliably and easily estimated with CV analysis—using alternative methods such as analysis of NMDA:AMPA ratio, paired-pulse ratio, or quantal analysis in parallel with CV analysis generally give rise to the same interpretation (Reid and Clements, 1999; Sjöström et al., 2007). Modern and more direct methods based on optical activation or readout are especially attractive alternatives (Jensen et al., 2017, 2019; Durst et al., 2019; Ellis-Davies, 2019; MacDougall and Fine, 2019; Mitchell et al., 2019; Padamsey et al., 2019), since they in many cases are virtually free of assumptions. Still, all methods come with their own advantages and caveats, e.g., analysis of paired-pulse ratio may erroneously suggest presynaptic expression for NMDA-only silent synapses that undergo postsynaptic expression (Poncer and Malinow, 2001), glutamate uncaging can necessarily only explore postsynaptic expression, and dyes used with optical methods may distort plasticity mechanisms by buffering calcium (MacDougall and Fine, 2019). It therefore remains important to use several methods in parallel. Classical CV analysis is one method that is both straightforward and inexpensive to use.

Here, we have listed a set of key pitfalls and shortcomings of the CV analysis method, which we have also illustrated in the form of simple downloadable computer simulations (see GitHub link in section “Materials and Equipment”). We have also provided a number of straightforward solutions for the most obvious issues. From this simple guide, it should be clear that CV analysis is a powerful and easy-to-use method, especially when combined with other approaches such as analysis of paired-pulse ratio or NMDA:AMPA ratio (Watt et al., 2000, 2004; Sjöström et al., 2007).

## Data Availability Statement

The raw data supporting the conclusions of this article will be made available by the authors, without undue reservation, to any qualified researcher.

## Ethics Statement

The animal study was reviewed and approved by the Montreal General Hospital Faculty Animal Care Committee (MGH FACC).

## Author Contributions

JB and AW carried out the experiments. SL carried out the mathematical derivations. PS wrote the custom software. JB, PS, and AT wrote the manuscript with input from AW.

## Conflict of Interest

The authors declare that the research was conducted in the absence of any commercial or financial relationships that could be construed as a potential conflict of interest.
